# Comparing AI and human-generated health messages in an Arabic cultural context

**DOI:** 10.1080/16549716.2025.2464360

**Published:** 2025-02-18

**Authors:** Syed Ali Hussain, Ralf Schmälzle, Sue Lim, Nassir Bouali

**Affiliations:** a College of Communication, University of Sharjah, Sharjah, UAE; b Department of Communication, Michigan State University, East Lansing, MI, USA

**Keywords:** Road safety messaging, generative AI, message evaluation, United Arab Emirates, Large Language Model (LLM)

## Abstract

**Background:**

AI is rapidly transforming the design of communication messages across various sectors, including health and safety. However, little is known about its effectiveness for roughly 420 million native Arabic speakers worldwide.

**Objective:**

This study examined characteristics of AI vs. human-generated road safety messages for a potential roadside billboard campaign in the United Arab Emirates.

**Method:**

The study includes a computational analysis and an online evaluation with 186 participants from the United Arab Emirates (UAE), comparing messages generated by AI with those created by humans. To achieve this, an AI model (GPT-4) was utilized to generate 15 road safety messages, while three human experts created another set of 15 messages. Computational text analysis was employed to examine these messages, followed by an online study in which human participants evaluated all messages based on message clarity and message quality.

**Results:**

The computational analysis revealed that AI-generated messages exhibited more positive sentiment with no significant differences in terms of readability/text difficulty. Participants evaluated both AI- and human-generated messages highly in terms of message quality and clarity, but human-generated messages were rated as slightly and significantly higher in terms of clarity.

**Conclusion:**

These results add to a rapidly growing body of research demonstrating that AI-generated messages can augment public communication campaigns and point towards the need to assess how diverse, international audiences respond to AI-generated content.

## Paper Context

- **Main Findings:** The study results show that AI-generated health messages exhibit more positive sentiment and are rated slightly lower in clarity compared to human-generated messages but are similarly rated in quality.
- **Added Knowledge:** The study demonstrates AI’s potential to generate culturally sensitive messages for public communication in a highly diverse Arabic-speaking population.
- **Global Health Impact for Policy and Action:** The study provides evidence of the feasibility of using AI-generated content to enhance health campaigns globally, emphasizing the importance of integrating cultural and linguistic adaptability into AI-driven public health messaging.

The emergence of Artificial Intelligence (AI) marks a significant turning point in how communication campaign messages are designed and evaluated. To date, most research examining AI-generated messages has focused on the US, Europe, China, and India, as they are major players in AI model development. However, it is clear that the messages generated by AI models are disseminated among worldwide audiences, thus highlighting the need to study them in other countries and among audiences who may differ from the aforementioned nations/cultures. Creating a health communication campaign for diverse population requires designing messages in multiple languages while ensuring that they are comprehensible, effective, and acceptable across cultural groups, and demographics. This challenge is the central topic of the current paper, which examines the potential of AI for road safety message generation in the culturally and linguistically diverse contexts. Specifically, this paper examines AI’s potential in creating effective messages for potential road safety campaigns in the context of the United Arab Emirates (UAE), one of the world’s most ethnically and culturally diverse countries [1].

This paper is organized as follows. First, we discuss AI methods for message generation and review related empirical studies and theoretical challenges. Next, we address the need for road safety messages that meet quality standards. We then focus on the issue of texting and driving, a risky behaviour in the UAE that public communication campaigns can address. Finally, we present the study’s method, results, and discussion.

## Background

### The rise of artificial intelligence for message generation and current challenges

As of 2024, AI is increasingly used in various areas of persuasive communication, including safety and health messages, advertising, and politics. Given that public communication campaigns require effectively conveying information to audiences, which relies heavily on language [2,3], this development stands to massively influence the theory and practice of campaigning.

Current large language models (LLMs) are already capable of generating communication content to inform (i.e. raise awareness and convey factual knowledge) and influence [4–7], and a swiftly growing body of research deals with using AI for message generation across virtually all communications contexts. For instance, AI has been used to generate advertisements, political slogans, short tweets, health messages, educational summaries, and messages for dozens of other special-interest domains [8,9]. Despite remaining challenges, generative AI is quickly transforming how communication content is being created, embodying the previously mythical ‘muse in the machine’ [10] that can come up with new messages almost instantly and with great variety.

Undoubtedly, the few studies published to date have barely scratched the surface of this new nexus between communication and generative AI. Rather, a great deal of communication research is needed to clearly understand the capabilities (e.g. whether AI-generated messages differ structurally and how they are perceived [11,12] and limitations or risks of AI systems (e.g. the risk of hallucinating false content [13]), how users respond to them (e.g. whether it matters that messages are labelled as AI-generated [4]), and so forth. Based on the still limited, but rapidly growing number of studies, it seems that AI-generated messages are generally evaluated as acceptable if not better than human-generated comparison messages [5–7,12–14].

### The potential of AI-based health message design for non-english-speaking audiences

Within this context, one challenge for AI is designing messages in multiple languages. Most AI systems are trained in English. Other languages are substantially less prominent. Although the field of multilingual AI has made significant progress and the large foundation language models master multiple languages, they are still most proficient in English, and the eventual negative consequences of their English advantage are not well understood. About applied linguistics for health messages, this matters because communicators are supposed to be unbiased. Theoretically, one would assume that a given health message should be equally effective in English as in another language. For instance, the simple fact-statement ‘smoking kills’ can be expressed in many different languages, such as Russian: ‘Курение убивает’, Spanish: “Fumar mata.’, French: “Le tabac tue.”, German: “Rauchen tötet.”, Arabic: “التدخين يقتل.”, Korean: ’흡연은 죽음을 가져옵니다.” However, not all health messages are of such a simple and strictly factual kind. Instead, health communicators often try to adapt their messages to characteristics of the audience, making them culturally sensitive.

### Road safety campaigns across the World and specifically in the UAE

Road safety campaigns play a crucial role in reducing traffic-related injuries and fatalities by targeting driver behavior [15,16]. Traditional campaigns use mass media, such as billboards and, to some extent, social media, to deliver brief messages where drivers are likely to see them. Research indicates these campaigns can improve behaviors like seatbelt use and reduce speeding or texting when messages are clear, relevant, and repeated enough [17]. However, ensuring these messages resonate with diverse audiences and lead to lasting change remains challenging. The development of effective safety communication is essential, especially with new technologies like AI offering potential for personalized messaging.

In the UAE, road safety is a major concern. Like the U.S., the UAE is car-centric, with rapid urbanization in cities like Dubai contributing to traffic safety issues [18]. In 2022, road injuries increased by 15%, with 65% of deaths and 57% of injuries caused by distracted driving, tailgating, driving under the influence, and negligence [19].

Crafting messages for the UAE requires careful consideration of its cultural diversity. With over 90% of its population being immigrants, the country has a wide variety of languages, religions, and socio-economic backgrounds [20]. Additionally, the driving population’s attitudes toward risk, safety, and traffic laws differ significantly from those in the U.S. or Europe. Taken together, the creation of road safety messages may not translate in a one-to-one fashion from US or European contexts. In the case of human message generation, this could be addressed by ensuring that message creators, focus groups, or copy testing/message pretesting are done with appropriate representation from local experts or audiences. However, in the case of using AI to generate messages, it remains an open question whether AI-generated messages would be on par with human-generated messages, as recent work using US-based LLMs in US-samples suggests [12,14,21].

## The current study

There is a need to examine the capabilities and limitations of generative AI, particularly in contexts outside well-studied Western settings. While generative AI has been explored in areas like marketing, health communication, and political messaging, we found no studies specifically addressing road safety. Given the societal importance of road safety and the likely future use of generative AI in this field, our objective was to assess AI-generated road safety messages. Specifically, we set out to compare AI-generated messages and human-generated messages. For this purpose, we used an AI-model (GPT4) to generate 15 road safety messages and had 3 human experts generate 15 messages. We then used computational text analysis to examine these messages and conducted an online study in which human participants evaluated all messages in terms of message clarity and message quality.

## Method

All analyses were conducted in python, and we document the analyses via reproducible Jupyter notebooks (https://github.com/nomcomm/uae_billboard_ai). Statistical analyses were also run in JASP program.

### Generation of human and AI-crafted road-safety messages

Human message generation process: First, we analyzed and discussed the issue (situation analysis) and audience (audience analysis), with a focus on the diversity in UAE. Next, we evaluated existing campaigns (previous campaign analysis) in the UAE and noticed a large number of multilingual messages, in both English and Arabic language, mostly presented side-by-side. The first three authors reviewed existing health campaign messages for road safety and came up with 15 messages each. Example messages include, ‘In a split second, you could ruin your future, injure or kill others, and tear a hole in the heart of everyone who loves you.’ From a pool of 45 message candidates, the authors then selected 15 messages for the study.

AI message generation process: For the AI-generated messages, we used the ChatGPT interface and prompted it to generate 15 messages for an intended roadside billboard campaign in the UAE (for details see Supplementary Materials). The authors then went through the messages and confirmed that they were appropriate for the study’s purpose.

**Figure 1.**
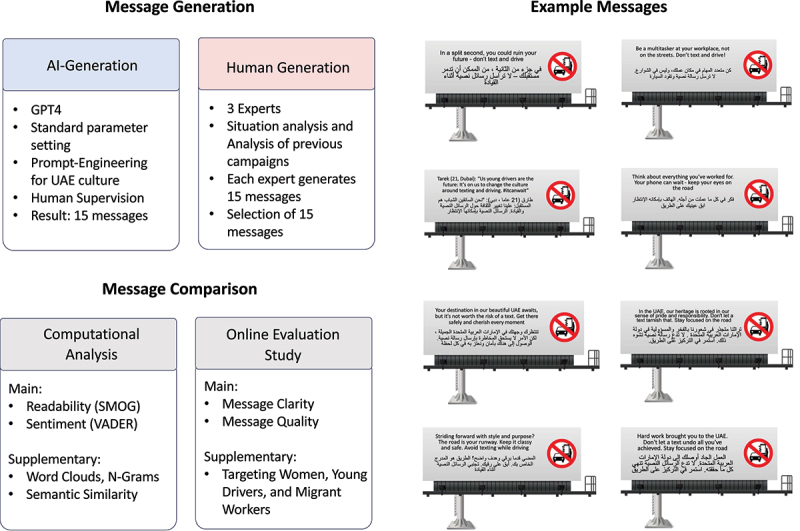
Study overview. ai-generated and human-generated billboard messages were created and compared via computational methods and human evaluations. The right panel shows 8 example messages as they were shown along with Arabic translations on images of billboard stands (see Supplementary Materials for the entire list).

Message translation and cultural appropriateness: We ensured that human-generated messages incorporated Arabic linguistic norms, cultural values (family, responsibility, and religious principles), and metaphorical expressions. AI-generated messages were prompted with cultural cues and reviewed by a native Arabic speaker. Additionally, messages were presented bilingually to reflect the UAE’s multilingual landscape, and the participant pool included a diverse mix of Emiratis and Arabic-speaking expatriates. Once the messages were generated and selected, a native speaker translated all messages into Arabic. The final messages were then put on a pictorial billboard stand (Figure 1), showing both the English as well as the Arabic language version of each of the 30 road safety messages.

### Computational comparison

To examine and compare characteristics of AI- and human-generated messages, we inspected word-cloud visualizations of the messages and assessed word frequencies (n-grams). Next, we analyzed per-messages text difficulty scores via the SMOG method [22,23] and per-message sentiment scores via the VADER package [24]. All data and Python scripts used to reproduce these data are available at (https://github.com/nomcomm/uae_billboard_ai). In brief, *SMOG (Simple Measure of Gobbeldygok*) is a readability statistic that is widely used in healthcare. It is similar in principle to the famous Flesch/Kincaid reading ease metrics, which gauge the number of years of education required to understand a text. The SMOG-index is calculated via the python package Textacy, which is a wrapper around the popular spaCy package for natural language processing. *VADER* (*Valence Aware Dictionary and sEntiment Reasoner*), on the other hand, is a python-based package that uses a lexicon in combination with rule-based algorithms to quantify text sentiment in messages. Thus, we submitted each message – human or -AI-generated – to the SMOG and VADER-functions, yielding one computational score for each message and each measure.

### Human evaluation (online study)

#### Participants

A total of 205 respondents from the UAE completed the study, which was approved by the local ethics review board. The study targeted a diverse group of participants, including Emirati nationals and expatriates, to reflect the UAE’s multicultural demographic. Recruitment was conducted through university networks, in-person announcements, and flyers, with two authors introducing the study in undergraduate and graduate classes. Interested individuals accessed the survey via Qualtrics or a QR code. Given that over 90% of the UAE’s population comprises expatriates [20], the sample was designed to include both Emiratis and non-Emirati Arabic speakers. We discarded responses from participants who finished in less than 200 seconds or provided incomplete responses, leaving 186 participants (49 self-identified males, 127 females, *m*_*age*_ = 21.9 years; *sd* = 4.9). 50% of the sample had UAE nationality, and all spoke either English or Arabic, and the majority had at least basic command of both languages. The final sample consisted of 50% Emirati nationals, with the rest representing Arabic-speaking populations from Egypt, Jordan, Saudi Arabia, Iraq, and Syria. This distribution aligns with UAE demographic reports, which highlight the significant presence of Arabic speakers from the region [18]. Additional details on participant demographics, sub-audiences, and their responses to specific messages are reported in the *Supplementary Materials*.

#### Procedure

The 30 candidate messages were displayed in random order, using the online survey platform Qualtrics. Thus, participants saw all messages but could not know whether they were generated by AI or humans. To increase realism as a formative evaluation study for a potential road safety campaign, we displayed all messages on a photograph of a billboard. Participants evaluated all messages in terms of perceived message clarity and perceived message quality (asking for Clarity: *‘Please evaluate the following message in terms of whether it is clear and easy to understand.’*; and for Quality: *‘The content and quality of this message is designed to promote safe driving. Please indicate how much you agree*.’). Participants’ answers were collected using a 5-point Likert-scale with response options ranging from very clear to very unclear (for clarity) and from strongly agree to strongly disagree (for quality). The survey also asked questions about demographics, driver status, and language competency.

### Data analysis

Analyses were conducted in python and via Jupyter notebooks [11] and the JASP [25] software package. All data and scripts are available at (https://github.com/nomcomm/uae_billboard_ai). We compared computational metrics of readability and sentiment between AI- and human-generated messages via independent sample *t-*tests. Data from the online study of message evaluations were downloaded, cleaned, and averaged for each message as well as by source categories (generated by humans or AI, respectively). To test for differences between AI- and human-generated messages, we computed linear mixed effects analyses for each measure, which allows modelling the variance by individual messages [26] as well as participants.

## Results

### Computational comparison

**Figure 2.**
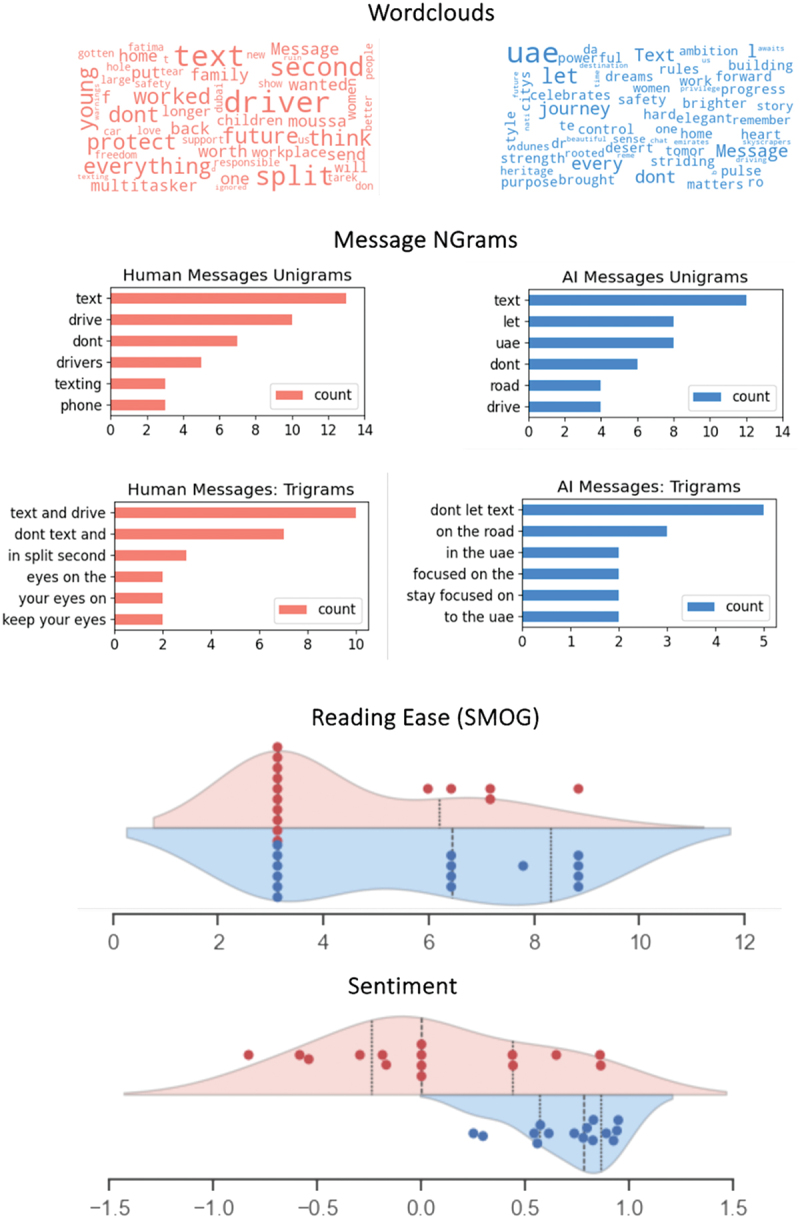
Examining the billboard messages with NLP-analytic tools reveal similarities and differences between human- and AI-generated text.

Computational analyses were performed to compare metrics of word frequency, reading ease, sentiment, and semantic similarity or AI- and human-generated road safety messages. Results revealed that the AI- and human-generated messages are comparable in terms of word frequency statistics and reveal expectable choices of words related to safety and distraction (Figure 2) The metrics of reading difficulty (SMOG score) have comparable distributions between AI- and human-generated content and did not differ in terms of their means (*m*_*readability:human*_  = 4.46, *sd* = 1.96; *m*_*readability:AI*_ = 5.84, *sd* = 2.39; *t(28)* = 1.68; *p* = 0.10). However, with regard to message sentiment, the AI-generated messages had a more positive sentiment overall (*m*_*sentiment:human*_  = 0.04, *sd* = 0.5; *m*_*sentiment:AI*_ = 0.7, *sd* = 0.21), and this difference is statistically significant (*t(28)* = 4.52; *p* < 0.001; see *Supplementary Materials* for further details).

### Comparing evaluations of message clarity and quality

Analysis of the human message evaluations revealed that all messages were judged around 4 on a 5-point scale (Table 1). Nominally, the human-generated messages have a slightly higher group mean compared to AI-generated messages, both in terms of perceived clarity and quality (*m*_*human:clarity*_ = 4.08, *m*_*AI:clarity*_ = 3.89; *m*_*human:quality*_ = 4.12, *m*_*AI:quality*_ = 4.04). However, the AI-generated messages were less variable (i.e. more consistent compared to the human-generated ones, which often include the best and worst messages from the pool (*sd*_*human: clarity*_= .28, *sd*_*AI: clarity*_ = .12; *sd*_*human: quality*_= .31; *sd*_*AI: quality*_= .14).

**Table 1.** Average evaluations of human- and AI-generated Road Safety Messages in terms of clarity and quality.

	*Human-generated Messages*	*AI-generated Messages*	*Statistics* *(LME)*
Message Clarity	*4.08* *(.28)*	*3.89* *(.12)*	*F*_*Source: Clarity*_ *= 5.09* p = .03 *
Message Quality	*4.12* *(.31)*	*4.04* *(.14)*	*F*_*Source: Quality*_ *= .78* *p = .38*

**Figure 3.**
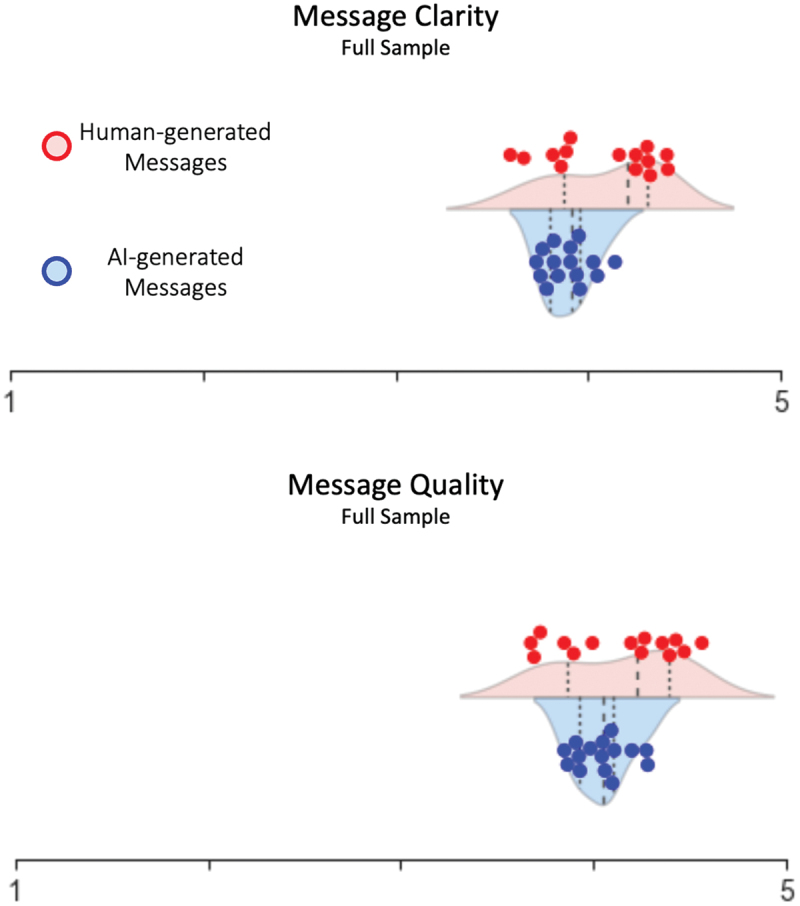
Average evaluations of individual human- and AI-generated Road Safety Messages in terms of clarity and quality. Scales range from 1–5.

Statistical assessment of these differences confirmed a difference between AI-generated and human-generated messages in terms of perceived message clarity (Figure 3), *F*_*Source: Clarity*_ (1, 33.19) = 5.09; *p* = 0.03, but there was no difference in terms of perceived message quality between AI- and human-generated messages (*F*_*Source: Quality*_ (1, 31.9) = 0.78; *p* = 0.38). Additional data and results are presented in the Supplementary Materials.

## Discussion

This study compared human- and AI-generated road safety messages in the context of the UAE using computational text analyses and human evaluations of message clarity and quality. The results from the computational analysis show that AI-generated messages use similar words, focus on similar topics, and they achieve similar scores in terms of text difficulty. Interesting observations from this stream of analyses were that AI-generated messages have higher sentiment. This could be explained based on how LLMs are trained based on filtered content and reward modelling via reinforcement learning from human feedback [27]. Another interesting result was that despite our familiarity with the immense knowledge stored in LLMs, we were still surprised by its relative ability to get involved with the cultural context of the UAE, and to generate messages that honour this context. The study aligns with studies that advocate for culturally sensitive messaging in communication campaigns [28].

Results from the human evaluation study showed that human-generated messages were rated slightly higher for perceived clarity than AI-generated ones, but this difference was statistically significant with a small effect size. There was no significant difference in perceived quality between human- and AI-generated messages, aligning with previous research where AI messages were rated well [4,11,12,29], except when participants knew they were AI-generated [4]. Generally, both human- and AI-generated messages were rated high quality (~4 on a 5-point scale), suggesting that they were all relatively acceptable. We believe that human-generated messages may have been exhibited more clarity due to their cultural relevance, emotional appeal, and natural linguistic variation. While AI-generated messages were likely more linguistically correct, human-created messages contained culturally embedded expressions, idioms, and localized references that resonated more with participants as discussed by Donia and Shaw [28]. Additionally, the emotional depth of human messages, particularly those incorporating personal and community-centered narratives, may have resulted in stronger engagement and comprehension since emotional connections enhance information retention [2]. Also, human messages exhibit more natural variations in sentence structure and word choice, making them more intuitive and relatable, whereas AI-generated texts, despite their grammatical accuracy, may have seemed more robotic due to algorithmic preferences and repetitive phrasing.

Of note, as discussed more extensively in the Supplementary Materials, we also looked for but found no significant effects of messages targeting sub-audiences, comprised specifically migrant workers, young drivers, and women drivers (who are all groups with a special status in the UAE in this context). This might be due to the generally weak effects of message tailoring/targeting, especially with short, clear billboard texts. Future research could explore more potent message manipulations, such as imagery or symbols, to study targeting in the Middle East. However, it should be considered that billboards as a mass communication device (i.e. a medium that is passed by many drivers from the targeted and non-targeted audiences) would have to be tested to not only boost potential targeting effects, but also minimize unintended consequences.

### Implications

This study highlights the potential for AI in message design, particularly in the MENA region, where AI-generated messages could significantly impact public communication for over 420 million Arabic speakers across 22 countries. Developing AI-designed messages can be cost-effective, allowing better allocation of public resources, but it also requires developing guardrails and safety standards [13].

Our study also underscores that while AI has promise as a message creator, designing AI-driven messages for the Middle East requires consideration of key cultural factors [28]. It is encouraging that the AI-generated messages were generally successful in meeting at least basic quality standards. This suggests that the implicit cultural knowledge that AI systems have absorbed is sufficient – despite being mostly based on western training data – to design messages that can address multicultural audiences.

### Limitations and avenues for future research

Like all research, this study has limitations. First, future work should expand the range of evaluation measures: The computational metrics, readability and sentiment, are both clear and relevant, but they represent only a subset of the dimensions along which messages generated by AI vs. humans could differ. For instance, it has been shown that AI is prone to use certain words more often, to have a more predictable syntactic structure (as a result of the LLM’s next-word-prediction operation principles), and our work underscores a slight positivity bias. More work is needed to analyze specific messaging strategies and attributes, such as the use of narratives, first-person perspective, or appeal types. With only 15 messages in each group, this was not feasible for this study, but it will become an option as more and more messages are going to be created via AI. Next, moving from computational analysis to the human online evaluation study, the current used single-item measures, which are efficient, reduce burden, and have been promising in related fields [30,31]. However, one could gain additional insights by using multi-item scales of concepts like perceived message effectiveness or argument strength [32].

Perhaps most importantly, it will be important to learn about the messages’ actual effectiveness rather than more or less elaborate pretesting indicators. In particular, a natural extension emerges if one considers that road safety messages are typically shown ‘in context,’ such as on billboards seen by drivers, which increases the chances of actual behavior change. To detect such behavioral effects accurately [33], it is necessary to measure message reception while people are driving, rather than through online surveys and via self-report. Future research could thus use field studies to assess their impact or perhaps rely on ecologically more valid presentation contexts like virtual reality [34].

While computational analysis showed that AI-generated messages were comparable in readability to human-generated messages, their slightly lower clarity ratings could be partially attributed to subtle linguistic and cultural mismatches. Even though, GPT-4 performed reasonably well in generating messages for our study, future research should explore AI models explicitly trained on Arabic-specific datasets.

In terms of sampling, while a sample size of 186 may not fully capture the diversity of all Arabic-speaking populations in the UAE, it aligns with prior research on message evaluation, where samples of 150–200 participants have been sufficient to identify significant patterns in perception and clarity [30–32]. To ensure internal validity, the survey included randomized message presentation to mitigate order effects, and both AI- and human-generated messages were evaluated within the same participant pool. However, the study acknowledges limitations, particularly in representing the full range of Arabic dialects, socio-economic backgrounds, and literacy levels across the UAE and MENA region. Future research could employ larger, stratified samples to enhance external validity by considering variations in age, education, income, and driving experience. Additionally, while this study focused on written message clarity, future work could explore real-world testing, such as displaying messages on digital billboards, to assess behavioral impact. Methods like eye-tracking or response-time analysis may also offer deeper insights into message effectiveness across diverse audiences.

### Future implications and next steps

To improve AI-generated Arabic content, future efforts should focus on developing Arabic-centric AI models trained on high-quality, diverse corpora that include both Modern Standard Arabic (MSA) and regional dialects. Existing models should be further refined to better capture linguistic nuances and cultural context. Additionally, AI’s cultural context awareness can be enhanced by integrating adaptive prompt tuning based on regional and cultural factors, ensuring AI-generated messages are evaluated by native Arabic speakers and cultural experts before dissemination.

The use of localized idioms, proverbs, and culturally significant themes can further improve message clarity and resonance. To address ethical concerns, developers should establish guardrails to prevent Western-centric biases from influencing Arabic-language AI-generated messages. AI-generated health and safety content should be co-designed with Arabic-speaking public health experts, linguists, and communication scholars to ensure alignment with social, religious, and linguistic expectations. Beyond linguistic improvements, AI tools must integrate cultural intelligence to generate messages that are both emotionally resonant and socially acceptable. This can be achieved through human-AI collaboration, leveraging AI’s efficiency with human expertise to create persuasive and locally relevant messages. Additionally, interactive learning and feedback mechanisms should be implemented to fine-tune AI models based on real-world audience feedback, particularly for public health and safety campaigns.

### Summary and conclusions

This study examined AI- and Human-generated road safety messages in the context of the UAE AI-generated messages exhibit more positive sentiment but are rated as slightly less clear than human-generated messages. However, both types were rated highly overall. These findings highlight AI’s potential to enhance public communication campaigns. Future research should explore how AI-generated content resonates with diverse international audiences and further refine its clarity and cultural adaptability.

## References

[1] Thomas S. English in the united Arab emirates. World Englishes. 2023;42:344–10. doi: 10.1111/weng.12560

[2] Schiavo R. Health communication: from theory to practice. Hoboken (NJ): John Wiley & Sons; 2013.

[3] Harrington NG. Persuasive health message design. Oxford (UK): Oxford Research Encyclopedia of Communication; 2016.https ://oxfordre.com/communication/display/10 .1093/acrefore/9780190228613.001.0001/acrefore-9780190228613-e-7?d=%2F10 .1093%2Facrefore%2F9780190228613.001.0001%2Facrefore-9780190228613-e-7&p=emailA4rlwFW9N.NiE&utm_source=chatgpt.com

[4] Lim S, Schmälzle R. The effect of source disclosure on evaluation of ai-generated messages: A two-part study. Comput Hum Behav Artif Humans. 2024;2. 100058. doi: 10.1016/j.chbah.2024.100058

[5] Breum SM, Egdal DV, Mortensen VG, et al. The persuasive power of large language models. Proceedings of the International AAAI Conference on Web and Social Media. Vol. 18, 2024. p.152–163. doi:10.48550/arXiv.2312.15523

[6] Karinshak E, Jin Y. AI-driven disinformation: A framework for organizational preparation and response. J Commun Manag. 2023;27:539–562. Available from doi:10.1108/jcom-09-2022-0113

[7] Burtell M, Woodside T. Artificial influence: An analysis of AI-driven persuasion. arXiv preprint. 2023. arXiv:2303.08721.

[8] Bubeck S, Chandrasekaran V, Eldan R, et al. Sparks of artificial general intelligence: Early experiments with GPT-4. arXiv preprint. 2023. arXiv:2303.12712.

[9] Borges AF, Laurindo FJ, Spínola MM, et al. The strategic use of artificial intelligence in the digital era: Systematic literature review and future research directions. Int J Inf Manag. 2021;57:102225. doi: 10.1016/j.ijinfomgt.2020.102225

[10] Gelernter D. The muse in the machine: computerizing the poetry of human thought. New York City (USA): Simon and Schuster; 2010.

[11] Kluyver T, Ragan-Kelley B, Pérez F, et al. Jupyter notebooks: a publishing format for reproducible computational workflows. In: Loizides F, Schmidt B, editors. Positioning and power in academic publishing: players, agents and agendas. IOS Press; 2016.

[12] Schmälzle R, Wilcox S. Harnessing artificial intelligence for health message generation: The folic acid message engine. J Med Internet Res. 2022;24:e28858. doi: 10.2196/28858 35040800 PMC8808340

[13] Kreps S, McCain RM, Brundage M. All the news that’s fit to fabricate: ai-generated text as a tool of media misinformation. J Exp Polit Sci. 2022;9:104–117. doi: 10.1017/XPS.2020.37

[14] Lim S, Schmälzle R. Artificial intelligence for health message generation: An empirical study using a large language model (LLM) and prompt engineering. Front Commun. 2023;8:1129082. Available from doi:10.3389/fcomm.2023.1129082

[15] Elliot B. Road safety mass media campaigns: A meta-analysis. Report No.: cR 118. Canberra: Federal Office of Road Safety; 1993.

[16] Phillips RO, Ulleberg P, Vaa T. Meta-analysis of the effect of road safety campaigns on accidents. Accid Anal Prev. 2011;43:1204–1218. doi: 10.1016/j.aap.2011.01.002 21376920

[17] Wundersitz L, Hutchinson T, Woolley J. Best practice in road safety mass media campaigns: A literature review. Soc Psychol. 2010;5:119–186.

[18] UAE Govt Portal. Population and demographic mix. 2022. Available from: https://u.ae/en/information-and-services/social-affairs/preserving-the-emirati-national-identity/population-and-demographic-mix

[19] Tesorero C. UAE records 343 road accident deaths in 2022, 10% less than previous year. Zawya. 2023. Available from: https://www.zawya.com/en/legal/crime-and-security/uae-records-343-road-accident-deaths-in-2022-10-less-than-previous-year-jmo6b2rz

[20] Siemund P, Leimgruber JR, editors. Multilingual global cities: Singapore, Hong Kong, Dubai. New York (USA): Routledge; 2020.

[21] Karinshak E, Liu SX, Park JS, Hancock JT. Working with AI to persuade: Examining a large language model’s ability to generate pro-vaccination messages. Proc ACM Hum-Comput Interact. 2023;7:1–29. doi: 10.1145/3579592

[22] McLaughlin G. SMOG grading–A new readability formula. J Read. 1969;12:639–646.

[23] Montani I, Honnibal M, Boyd A, etal. explosion/spaCy: v3.7.2. 2023. doi:10.5281/zenodo.1212303

[24] Hutto C, Gilbert E. VADER: A parsimonious rule-based model for sentiment analysis of social media text. In: Proceedings of the International AAAI Conference on Web and Social Media; 2014. p. 216–225. doi:10.1609/icwsm.v8i1.14550

[25] Love J, Selker R, Marsman M, et al. JASP: Graphical statistical software for common statistical designs. J Stat Soft. 2019;88:1–17. doi: 10.18637/jss.v088.i02

[26] Jackson S, Jacobs S. Generalizing about messages: Suggestions for design and analysis of experiments. Hum Comm Res. 1983;9:169–191. doi:10.1111/j.1468-2958.1983.tb00691.x

[27] Wolfram S. What is ChatGPT doing:… and why does it work? Champaign (IL): Wolfram Media; 2023.

[28] Donia J, Shaw JA. Co-design and ethical artificial intelligence for health: An agenda for critical research and practice. Big Data & Soc. 2021;8:20539517211065248. Available from doi:10.1177/20539517211065248

[29] Cox SR, Abdul A, Ooi WT. Prompting a large language model to generate diverse motivational messages: A comparison with human-written messages. In: Proceedings of the 11th International Conference on Human-Agent Interaction; Gothenburg, Sweden; 2023. p. 378–380.

[30] Allen MS, Iliescu D, Greiff S. Single item measures in psychological science. Eur J Psychol Assess. 2022;38:120–134. doi: 10.1027/1015-5759/a000699

[31] Bergkvist L, Rossiter JR. The predictive validity of multiple-item versus single-item measures of the same constructs. J Mark Res. 2007;44:175–184. doi: 10.1509/jmkr.44.2.175

[32] Noar SM, Bell T, Kelley D, etal. Perceived message effectiveness measures in tobacco education campaigns: A systematic review. Commun Methods Meas. 2018;12:295–313. doi: 10.1080/19312458.2018.1483017 31428217 PMC6699787

[33] Thaler RH, Sunstein CR. Nudge: the final edition. New Haven (CT): Yale University Press; 2021.

[34] Cho HJ, Lim S, Turner MM, et al. Eyes on VR: Unpacking the causal chain between exposure, reception, and retention for emotional billboard messages. bioRxiv. 2024. doi:10.1101/2024.07.19.604208

